# Positive aspects of negative data

**DOI:** 10.1016/j.jmccpl.2022.100016

**Published:** 2022-09-28

**Authors:** David Eisner

**Affiliations:** The University of Manchester, United Kingdom

**Keywords:** Reproducibility, Data, Rigor

There is currently widespread concern about how much of science is reproducible. The effects of lack of reproducibility are as obvious in cardiovascular research as in other areas. A notorious example concerns a body of work in the stem cell field where fraudulent preclinical work led to a clinical trial, the results of which had to be retracted [1]. As discussed elsewhere, this story has lessons for both scientific journals and funders [2], [3].

Fraud is one cause of a lack of scientific reproducibility but there are many others. Science is carried out by humans, and we all make mistakes. There are also issues relating to the design and statistical analysis of studies which make the results irreproducible [4], [5].

The purpose of this editorial and the associated issue of JMCC+ is to consider the extent to which the difficulty of publishing negative results contributes to the reproducibility problem. The problem can be illustrated by the following fictitious example. Imagine that ten laboratories are studying the effects of snake oil on the heart. To their intense disappointment, nine of these find no effect. The researchers rapidly lose interest in this area and their data are relegated to dusty filing cabinets. If they try to submit their work to journals, it will probably be rejected for being “negative” and not sufficiently “impactful”. In contrast, the tenth laboratory discovers that this oil restores the function of the failing heart. Their work is then published in the most prestigious journals, and brings the authors further funding and career advancement, leading to yet more publications. Eventually, these laboratory studies are translated into a clinical trial giving patients snake oil, with the risk of damage to them. In other words, the publication bias whereby positive results are more likely than negative to be published has skewed the scientific record with serious consequences. It is precisely to avoid this publication bias that clinical research demands that trials are preregistered and then published no matter what the outcome.

It is easy to understand how this bias has arisen. Readers will be much more interested in positive rather than negative results, in the same way as we would feel frustrated if the conclusion of a detective novel was that X was NOT the murderer, with no information as to who had “dunnit”. Given that journals compete for articles, and that the authors are attracted to journals with higher Impact Factors, requiring many citations, it is not surprising that most journals are leery of publishing negative results.

While discussing negative results, it is important to realize that these come in various flavours which will have to be handled differently. I can think of the following three types, but others may add to them.

1. “*Dump on*”. Many years ago, when I was a graduate student, Jon Lederer and I found that the Purkinje Fibres on which we were working were very robust so that when we had finished the original experiment, we would apply various compounds which we had come across while reading the literature. Many of these had no effect but some were more instructive. If we could track down and play back the FM tapes, then the former could contribute to collections of negative data. An obvious question is whether such negative data should be published in the form of traditional papers? An alternative would be to curate such negative results in a database. We already have databases for genes, proteins, taxonomy, and many other biological areas. I see no reason why there should not be one for negative data.
2. *Hypothesis disproving*. Sometimes negative results come from a study where the hypothesis predicts a result. Failure to find this result disproves the hypothesis. In principle, such results should be published. How important they are depends on the hypothesis. If it is widely held then the result may be of great interest. It must, however, be remembered that negative results can occur simply because of lack of precision in measurements. A power calculation is required to ensure that the experiment could have detected the change predicted by the hypothesis.
3. *Repudiating previous work*. This is the situation where authors cannot reproduce previously published work. It is essential that such work is published. In many cases, it is useful if the disagreement is discussed with the original authors in order to see whether differences arise from differences of experimental conditions. The danger is that this can be used to delay publication of critical work. It is worth considering whether a journal that publishes a paper should be obliged to publish a subsequent paper that repudiates it, provided that it is of satisfactory scientific quality? Related to this, how should we publish subsequent work on either side of the argument?

Finally, while this editorial has concentrated on negative results, there is an equally important issue about confirmatory data. Journals have traditionally not wanted to publish papers that reach the same conclusion as previous work. As a reader, I can understand this. Why should I want to read a paper if I “know” the result already? Of course, the reasons for publishing negative data also support confirmatory papers. We can feel more secure about a result which is obtained in more than one laboratory.

I end by applauding the initiative of JMCC+ to embrace the area of reproducibility and, in particular, publishing negative data.

## Declaration of competing interest

No conflict of interest.
